# ACE polymorphism in asthmatic patients

**DOI:** 10.1186/2045-7022-3-S1-P14

**Published:** 2013-05-03

**Authors:** Margarida Cortez, Andreia Matos, Joana Ferreira, Leonor Lopes, Angela Gil, Manuel Bicho

**Affiliations:** 1 CHLN-HSM, Portugal; 2 Lisbon Medical School, Genetic Department, Portugal

## Background

The aim of the this study was to analyse if there is an association between angiotensin converting enzyme (ACE) insertion/deletion (I/D) polymorphism (287 base pairs, on chromosome 17q23, intron 16 (rs4340)) with asthma severity. ACE plays a vital role in the renin-angiotensin-system (RAS) which regulates blood pressure by converting angiotensin I into a powerful vasoconstrictor angiotensin II, that also has an important role in airway remodeling and in inactivation of bradykinin and tachykinins. The signaling pathways, related with this polymorphism regulating the cytokine production by T cells, could induce a different Th profile modulating the immune response in asthma.

## Methods

Asthmatic patients: n=68; were compared with a control group of n=204 healthy blood donors. The insertion/deletion (I/D) polymorphism was determined by PCR- polymerase chain reaction. Control of asthma assessed by validated instrument (ACQ7 and PAQLQ). Statistical analysis was performed with PASW 18, establishing a significance level of p<0.05.

## Results

The mean age of the 68 asthmatics was 39.95±18.9 years; 42 females and 26 males; 66 Caucasians and 2 non-Caucasians; 57 atopic and 11 nonatopic. The mean age of the control-group (n=204) was 40.97±12.08 years; 69 females and 135 males. In asthmatics the frequencies of the D-Allele (ACE-D) is 0.647 and of the I-Allele (ACE-I) is 0.353; in controls: 0.868 and 0.132 respectively. There is statistical difference between these groups (p=0.008). Genotypes in the asthmatics- DD: 44.1%; ID: 20.6%; II: 35,3%; in control group- DD: 48%; ID: 38.8%; II: 13.2%. There is statistical difference between these groups (p=0.000). The II genotype was more frequent in the asthmatics when compared with controls being the risk associated 3.576 (CI 95% [1.883 – 6.799], (p=0.000). In asthmatics, there is no statistical differences in genotype frequencies (p>0.05) between: atopics and non atopics; controlled and uncontrolled asthma; males and females; and in the different age-groups.

## Conclusions

The role of ACE insertion/deletion (I/D) polymorphism, in asthmatic patients is a controversy risk factor to the severity of asthma, but we concluded that the II genotype is more prevalent in the asthmatics from this hospital-based population.

